# INVESTIGATING THE NEURAL AFFECTS OF 17 ALPHA-ESTRADIOL (17Α-E2) ON APOE4 PHENOTYPES

**DOI:** 10.1093/geroni/igac059.2849

**Published:** 2022-12-20

**Authors:** Tavia Roache, Christian Pike, Cassandra McGill

**Affiliations:** Howard University, Washington, District of Columbia, United States; USC, Los Angeles, California, United States; University of Southern California, Los Angeles, California, United States

## Abstract

Aging and APOE4 genotype are the primary risk factors for late onset Alzheimer’s disease (AD). Previous drugs trials for AD have focused on amyloid beta which has yielded minimal effects; hence a drug targeting aging could prove more efficacious. 17 alpha-estradiol (17α-E2) is an enantiomer of 17 beta-estradiol that has been shown to extend lifespan in male mice. We hypothesize that 17α-E2 will improve aging related phenotypes caused by the APOE4 genotype. We enrolled 10-month-old APOE3 or APOE4 targeted replacement mice and randomized them to either control or 17α-E2 diet for 20 weeks. At weeks 15 to 17, mice underwent various behavior assays and tissues were collected at the end of the 20 weeks. We observed behavioral improvement in APOE4 mice treated with 17α-E2 and asked whether this effect is associated with: (1) change in activity or anxiety, (2) change in neural stem cells, or (3) change in microglial activation. Activity levels but not neurogenesis of microglial burden were affected by APOE genotype and 17α-E2 treatment, suggesting a potential contribution to observed memory improvement in APOE4 mice treated with 17α-E2. Continued research on the neural effects of 17α-E2 pose potential benefits to mitigate the effects of aging.

